# Assessing Dietary Consumption of Toxicant-Laden Foods and Beverages by Age and Ethnicity in California: Implications for Proposition 65

**DOI:** 10.3390/nu17193149

**Published:** 2025-10-02

**Authors:** Shahir Masri, Sara Nasla, Denise Diaz Payán, Jun Wu

**Affiliations:** 1 Department of Environmental and Occupational Health, Joe C. Wen School of Population & Public Health, University of California, Irvine, CA 92697, USA; snasla@uci.edu; 2 Department of Health, Society and Behavior, Joe C. Wen School of Population & Public Health, University of California, Irvine, CA 92697, USA; dpayan@hs.uci.edu

**Keywords:** dietary survey, food questionnaire, contaminants, Proposition 65, consumption patterns, food policy

## Abstract

Background: Investigating human exposure to toxic contaminants through dietary consumption is critical to identify disease risk factors and health guidelines. Methods: In this study, we developed a cross-sectional online survey to collect information about dietary patterns and related food consumption habits among adults (age ≥ 18) and adolescents (ages 13–17) in Southern California, focusing on popular staple foods and/or those targeted most commonly under California’s Proposition 65 law for lead and acrylamide exposure. Results: Results identified root vegetables, rice, leafy greens, pasta/noodles, tea, juice, and seafood to be among the most heavily consumed foods by mass, while the daily intake of many foods such as stuffed grape leaves, tamarind/chili candy and herbs/spices varied by age and race/ethnicity, suggesting that many of Proposition 65’s pollution allowances may be exacerbating issues of health inequity and environmental injustice. Moreover, findings from this study indicate that the methods of exposure assessment often applied under Prop 65, especially relating to herbs/spices, are likely to underestimate single-day exposures, thus allowing unsafe products on the market without warning labels. Conclusions: Study outcomes are broadly relevant to environmental health and nutrition science, with particular relevance to public health practitioners and California’s Prop 65 regulators and other stakeholders.

## 1. Introduction

Investigating human exposure to toxic contaminants through dietary consumption is critical to identifying disease risk factors and health guidelines. While studies have examined food consumption patterns and disparities by race/ethnicity and/or socioeconomic status (SES), existing work examines broad categories (e.g., sweets, plant-based eating, etc.) instead of specific foods and ingredients and often overlooks exposure to food contaminants [1,2].

Several foods and beverages are known to disproportionately accumulate contaminants. Policies and guidelines at the local, national, and international level have therefore been enacted to help safeguard sources of food and drink; with examples including the U.S. Food & Drug Administration’s “action levels” set for arsenic and lead in juice, the European Commission’s “maximum levels” for contaminants in food, and the World Health Organization’s guidelines for drinking water [3,4,5]. In California, the Safe Drinking Water and Toxic Enforcement Act of 1986 (Proposition 65), enacted through a ballot initiative, requires businesses to warn residents about harmful chemical exposures. Prop 65 also requires the state’s Office of Environmental Health Hazard Assessment (OEHHA) to publish and maintain a regularly updated list of chemicals (~1000) known to cause cancer, birth defects and/or reproductive harm [6]. Consumer goods sold in California are subject to Prop 65, with some exemptions, and therefore must contain “clear and reasonable” warnings if containing elevated Prop 65-listed chemicals [7,8,9]. Listed chemicals include natural and synthetic chemicals such as heavy metals (i.e., lead, arsenic, cadmium) and organic contaminants (i.e., acrylamide, phthalates, pesticides). Exposure to such chemicals is associated with a range of adverse health outcomes.

In the case of lead exposure, health outcomes include asthma [10,11,12,13] and adverse neurological and cognitive outcomes in children [14,15,16,17], as well as pregnancy complications in women [18,19,20,21]. Similarly, cadmium has been associated with high blood pressure, hypertension, and osteoporosis [22,23,24], while arsenic has been linked with peripheral vascular and cardiovascular diseases in chronically exposed populations and to type II diabetes [25,26,27].

To protect against harmful exposures to chemicals in consumer products, California has published benchmarks known as “Safe Harbor” levels expressed in units of micrograms (μg) of exposure per day, which include Maximum Allowable Dose Levels (MADLs) to prevent reproductive effects and No Significant Risk Levels (NSRLs) to avoid a 10^−5^ lifetime risk of cancer. Each benchmark is meant to be applied universally, irrespective of age and/or body weight.

Exposure to contaminants through foods and beverages is an active area of Prop 65 enforcement. However, Prop 65 considers individual chemicals and food-related exposures in isolation without accounting for the potentially additive or synergistic impacts of chemical mixtures or cumulative exposures through multiple foods. Moreover, experts often use single serving size values listed on product labels to estimate average daily intake, which may underestimate exposure to pollutants—particularly for items like herbs and spices frequently used in tandem. Other questionable approaches stem from assumptions around non-edible foods (e.g., tea bags are only steeped for 3–5 min, limiting the leaching of contaminants), which are often derived from product labels and consumption instructions rather than population-based surveys [28]. Additionally, product-specific “naturally occurring” exemptions have arisen under Prop 65, which establish discrete pollution benchmarks that permit contamination (e.g., lead allowed in tamarind/chili candy) despite harmful exposures to consumers, especially children [29,30].

Herbs/spices, dietary supplements, seafood, and leafy greens are among the most commonly pursued food categories by Prop 65 enforcers when considering heavy metal pollutants [31]. Such pollutants may disproportionately accumulate in foods due to product manufacturing and distribution (e.g., contact with metal factory components and dust), natural uptake from soil, bioaccumulation through the food web (e.g., fish) [32,33,34], canned storage (e.g., metals leaching into foods from canning solder) [35,36,37], and cooking processes (e.g., high-temperature processes producing acrylamide and heterocyclic amines) [38,39,40]. According to the U.S. Food and Drug Administration (FDA), tea and juice are also concerning sources of lead [41,42,43]. Given regional differences in pollution emissions globally, product and/or ingredient origin may also influence contaminant levels. This is of particular concern in growing environments that are non-commercial (e.g., home gardening) and therefore not subject to government soil and product testing, especially where residential communities are near current or historical pollution sources (often consisting of low-income communities and those of color).

Given a paucity of population-based dietary intake data for some foods (i.e., dietary supplements, herbs/spices, and candies/chocolates), it can be challenging to estimate hazardous exposures. For example, while herb/spice intake may be associated with health benefits (e.g., improved cardiovascular and metabolic health), few studies quantify the cumulative intake of herbs/spices [44] to assess harmful exposures. In a study of Norwegian adults, Carlsen et al. (2011) calculated a median cumulative daily intake ranging from 1.6 to 2.7 g across the herb/spice category, while studies elsewhere document herb/spice intakes >10 g/day [45,46,47,48]. These studies suggest consuming individual herbs/spices may vary by an order of magnitude by region and race/ethnicity. Understanding differences in consumption patterns by race/ethnicity is therefore important given that certain foods can produce higher chemical exposure burdens. This is particularly relevant given public health research and messaging promoting the Mediterranean diet, plant-based eating, and increased consumption of foods like herbs/spices, dark chocolate and fish [44,49].

Despite prior health survey data and contemporary nutrition and dietary assessment measures, the ability to estimate food-related exposures is limited due to the exclusion of certain food categories. For example, the National Health and Nutrition Examination Survey (NHANES), a leading population-level continuous survey to evaluate the nutrition and health of adults and children in the U.S., has limited information about herbs/spices [50]. Moreover, food frequency questionnaires (FFQs) often exclude herb/spice intake, inadequately measure intake/use in food preparation, and require long participant recalls (e.g., 1 month) that may be inaccurate [51].

To address these gaps, we constructed and utilized a 1-week dietary survey (improving upon the 1-year recall of NHANES) to quantify portion sizes and consumption frequencies of key staple foods and beverages and those commonly targeted under Prop 65 (including twenty herb/spice categories) to estimate average daily intake and whether intake patterns differed by age and racial/ethnic characteristics among California adult and adolescent residents. We also examined behavioral patterns that affect food-related exposures, including consumption of home-grown foods and food preparation techniques, to inform research and food-related exposure assessment.

## 2. Materials and Methods

### 2.1. Study Design, Recruitment, and Sampling

A cross-sectional online survey was designed to collect information about dietary patterns and related food consumption habits among adults (age ≥ 18) and adolescents (ages 13–17) in Southern California. The survey focused on popular staple foods likely consumed in high quantities and/or those targeted most commonly under Prop 65 for lead and acrylamide contamination, as reported by the California Attorney General (although the concentrations of these contaminants and related exposures were not evaluated here) [52]. The survey was administered using Qualtrics (Provo, UT, USA) between 1 March and 15 June 2023. Survey recruitment methods included snowball sampling using local community contacts (e.g., university groups, non-profit organizations, etc.) who circulated the survey in English and Spanish via email listservs and social media platforms (e.g., Facebook, Instagram, etc.).

A total of 207 surveys were completed; however, we removed the following: 21 respondents who were either below the age threshold, did not reside in California, or had unreliable survey completion times (<3.38 min, corresponding to 1st percentile completion time). Median survey completion time was 8.15 min (Interquartile Range: 5.9 to 11.6 min). Following exclusion and data cleaning, N = 186 surveys were analyzed. Participants originated from Southern California, where counties mostly consist of White (26 to 50%), Hispanic/Latino (34 to 86%) and Asian (2 to 22%) residents [53].

### 2.2. Measures

Food consumption frequency measures were adapted from the NHANES FFQ (though improved upon by reducing recall period) with multiple-choice questions asking participants whether they consumed a specific type of food over the past 7 days [54]. For those who confirmed eating a specific food, they were then asked (multiple-choice format) to indicate the amount of that food they typically ate [55].

For certain foods, portion sizes were not evaluated. For example, beverages were pre-designated as one glass (5 oz) for wine and one cup (8 oz) for other liquids. Similarly, questions about powders were assumed to be a single serving, the average mass of which was estimated through the United States Department of Agriculture’s (USDA) FoodData Central [56].

Consumption frequency questions were included for 20 different herbs and spices (e.g., In the past 7 days, how often did you consume oregano?). To reduce respondent burden and align with FDA’s Reference Amounts Customarily Consumed (RACC) industry guidance document [57], only a single consumption quantity question was asked about herbs and spices (i.e., Each time you use herbs/spices, how much do you usually use?). For this food category, participants were also asked whether they used the single serving size labels on herb/spice product packaging to inform their quantities of consumption. Similarly, for tea, participants were asked about repeated tea bag usage. Participants were also asked about their cumulative water intake (e.g., Of the water you consume each day [including plain water and water added to make soups, coffee, juice mixes, smoothies, etc.], what proportion is unfiltered tap water?) and home-grown food intake (i.e., Of the food you consume, do you grow any of it in your home or yard?). Finally, participants were asked whether their homes were built before 1978 (the year lead paint was banned in the U.S.) in order to assess risks related to non-food lead sources. The full list of survey questions about food/beverage consumption, homegrown food, and housing age are included in Table S1 of Supplementary Materials.

Socio-demographic questions included gender, age, race/ethnicity, educational attainment, and household income. “Indian” and “Middle Eastern” groups were treated as distinct from the “Asian” racial/ethnic group (the latter referring only to East and Southeast Asia in this study) given the widely varying cuisines and ingredient profiles of traditional foods consumed by each group. Specifically, Indian cuisine is often characterized by its use of aromatic spices such as turmeric, coriander, cumin, and garam masala, along with its use of dairy products, such as yogurt, ghee, and paneer, and includes many vegetarian plates consisting of chickpeas and lentils. In contrast, Middle Eastern cuisine utilizes a more subtle blend of herbs and spices such as mint, parsley, and za’atar, along with ingredient such as olive oil, humus, and grape leaves, and makes greater use of meat products such as lamb, chicken, and beef (often in the form of grilled kebabs and shawarma). Cuisines originating from East and Southeast Asia, on the other hand, are known for their use of umami-rich ingredients, fresh vegetables, seafood, and noodles (often cooked using techniques such as stir-frying, steaming, and grilling). All three cuisines include rice as a central component of many dishes.

### 2.3. Data Management and Analysis

Responses were analyzed using SAS software, Version 9.4 [58]. Where consumption quantity options included ranges, responses were converted to the range midpoint (e.g., 1–2 cups converted to 1.5 cups). “Less than” responses were converted to one-half unit (e.g., <1 cup converted to 0.5 cups), while boundless “greater than” responses were assumed to be plus one unit (e.g., >3 cups converted to 4 cups). This approach was also used for consumption frequency (e.g., “2 to 4 times per week” converted to 3 times per week).

The USDA’s FoodData Central database was used to convert quantity values (e.g., cups, teaspoons, etc.) to a single metric (grams) [56]. “Consumption amount” was multiplied with “consumption frequency” for each food to yield a daily intake expressed in units of grams consumed per day (g/day) for each participant. When calculating summary statistics for consumers, participants reporting zero consumption (non-consumers) were removed from analysis. Such observations were retained for the “per capita” analysis.

To approximate a participant’s daily intake of herbs/spices, consumption frequency was first calculated for each herb/spice. This value was then multiplied by a constant of 0.83 tsp per eating occasion (EO), which was the average consumption quantity per eating occasion calculated across all participants for the entire herb/spice category. This yielded a volumetric intake per eating occasion for each herb/spice, which was multiplied by the density (g/tsp) of that herb/spice as reported by the USDA [56] to yield a mass intake value for each. Daily intake of each herb/spice was then summed for each participant to yield Total Herb/Spice daily intake.

Average consumption values (i.e., portion size, consumption frequency, daily intake) were compared between age and racial/ethnic groups, with differences between such statistics and the overall Group Mean (where n >1) presented below. Multivariate regression analyses were conducted separately for each food using age and race/ethnicity as independent predictors (reference group: White/Caucasian). These were the only two covariates examined due to sample size limitations and preexisting arguments that such sub-divisions should be accounted for under Prop 65. Statistical significance was assessed at the *p* < 0.1 and *p* < 0.05 levels.

## 3. Results

### 3.1. Descriptive Statistics

Table 1 presents socio-demographic characteristics of participants. Of N = 186 survey respondents, nearly two-thirds were female (62.9%) and 60.8% were adults. A majority identified as Asian/Pacific Islander (38.3%), followed by Latino/Hispanic (22.9%) and White/Caucasian (11.2%). About 40% reported a household income between $25,000 to $100,000, with ~20% reporting income below this range. Approximately two-thirds had a high school diploma or less, with the remaining third having completed at least some college. The majority resided in Orange County, California, with 80.5% residing in five cities including Irvine (22.8%), Placentia (20.1%), Yorba Linda (18.1%), Anaheim (13.4%), and Fullerton (6.0%).

### 3.2. Food & Beverage Intake Consumption Patterns

Table 2 presents consumption statistics of foods and beverages per capita and for consumers only. Foods consumed in greatest daily abundance per capita, on average, were root vegetables (83.9 g/day) (e.g., mashed potatoes, carrots, onions, etc.), followed by rice (52.6 g/day), leafy greens (33.6 g/day), and pasta/noodles (30.9 g/day). These foods were also consumed by the highest percentage (>80%) of participants. Among consumers only, the most abundantly consumed foods remained root vegetables (87.2 g/day) and rice (57.8 g/day), followed by non-fish seafoods (55.8 g/day) and stuffed grape leaves (44.5 g/day). Each showed a much higher daily intake when averaged across consumers compared to per capita.

Among consumers, stuffed grape leaves (consumed by only 6% of respondents), showed the highest portion size (242 g, about 6 stuffed leaves), followed by non-fish seafood (211.9 g), root vegetables (including fried potatoes), pasta/noodles, fried fish and other fried meat (95–130 g). Of participants who consumed homegrown food, most (60%) reported none of their root veggies or leafy greens were homegrown. Other homegrown fruits and vegetables were consumed 1–3 times per week by roughly half of home-growers.

**Figure 1:**
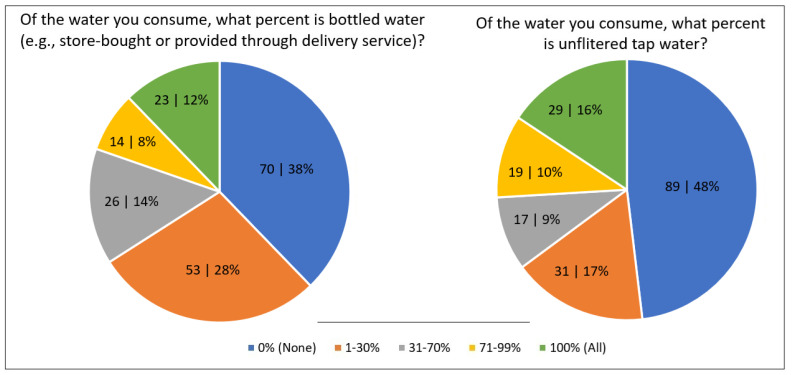
Distribution (n | %) of survey responses regarding water intake.

Among beverages, water was consumed in greatest daily abundance (1413 g/day) on both a per capita and consumers-only basis. Figure 1 presents the response distribution for water intake. For sources of water (including water for beverages, soups, etc.), two-thirds of participants said they consumed up to 30% of their daily water as store-bought bottled water or water provided by a delivery service, with 20% ingesting most (>70%) of their water from such sources. 1 in 4 (26%) said they consumed >70% of their daily water from unfiltered tap water.

Juice (72.4 g/day) and tea (70.2 g/day) were the second most abundantly consumed beverages per capita (~60% and 46% of participants, respectively). Among consumers, tea (151.7 g/day) and juice (120.5 g/day) intake amounted to roughly two-thirds and one-half a cup per day, respectively. Roughly one-third of tea users reported reusing their tea bags “always” or “sometimes” when drinking multiple cups of tea per day.

Among powdered foods, nutritional powder (e.g., protein powder) was consumed in greatest daily abundance per capita (6.8 g/day), followed by chocolate beverage mix (2.4 g/day) and traditional beverage powder (0.3 g/day) (e.g., matcha powder); each consumed by 22–33% of participants. Nutritional powder was similarly consumed in greatest daily abundance (24.3 g/day) among consumers, followed by chocolate beverage mix (10.8 g/day) and traditional beverage powder (0.8 g/day).

The food and/or beverage category consumed most frequently by consumers (besides water) was tea (0.65 EO/day, or 4.5 EO/week), followed by rice (0.60 EO/day) and nutritional powder (0.58 EO/day). Root vegetables, leafy greens, nuts/seeds, and chocolate beverage powder were also consumed at high frequencies (~0.5 EO/day) by consumers.

Regarding candies/snacks known to contain high lead, chocolates were consumed by ~80% of participants at a frequency of nearly every other day (average portion size: 30.0 g), with a mean daily intake of 12.2 g/day among consumers. Tamarind/chili candy was consumed by 24.1% of participants at a frequency of roughly once per three days (mean daily intake: 6.4 g/day).

### 3.3. Food & Beverage Intake Consumption Patterns by Age Category

**Figure 2:**
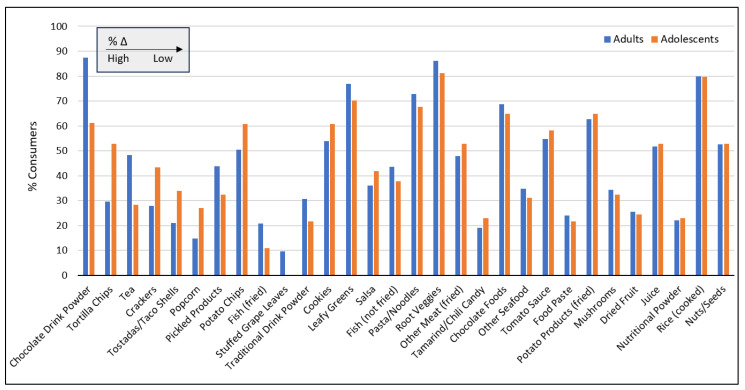
Percent of adults and adolescents consuming various foods and beverages, ordered left-to-right based on %Δ (difference in percentages between adults and adolescents).

Figure 2 presents the percentage of participants consuming foods and beverages by age category. The greatest differences in the proportion of consumers among each age group was for chocolate beverage powder, consumed by 87.5% of adults and 61.1% of adolescents (Δ = 26.4%), followed by tortilla chips, consumed disproportionately by adolescents (Δ = 23.1%). Considering the ratio of adult to adolescent consumers, stuffed grape leaves showed the highest discrepancy (consumed exclusively by adults), followed by fried fish (consumed by nearly twice the percentage of adults than adolescents), and popcorn and tortilla chips (consumed by 82.8% and 78.3% more adolescents compared to adults, respectively). In general, adolescents reported substantially higher portion sizes and daily intakes, with an average percent difference of +17% and +24%, respectively, across all foods.

Using multivariate regression analyses (Table 3), rice (*p* < 0.05), tostadas/taco shells (*p* < 0.05), juice (*p* < 0.05), tea (*p* < 0.1), crackers (*p* < 0.05) and popcorn (*p* < 0.05) showed statistically significant differences in daily intake when comparing adults and adolescents. Adolescents consumed about 30–80% higher daily quantities of each food, with rice showing both the highest mass difference (Δ = 41.4 g) and percent difference between age groups. Rice showed the highest variance explained (r^2^ = 0.27), followed by salsa, taco shells/tostadas, and grape leaves (each~r^2^ = 0.18). 

### 3.4. Food & Beverage Intake Consumption Patterns by Race/Ethnicity

**Figure 3:**
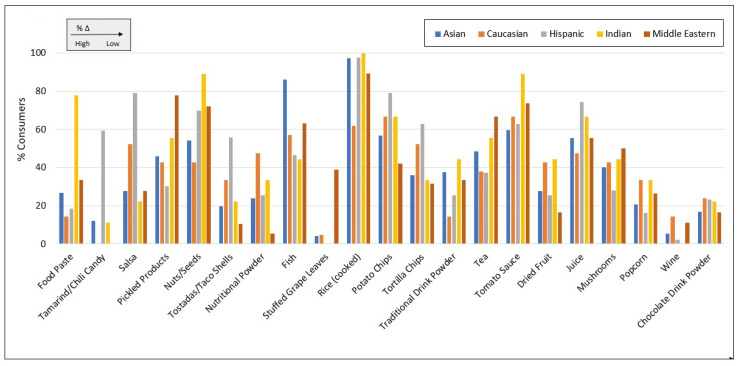
Percent of racial/ethnic groups who report consuming various foods and beverages, ordered left-to-right based on %Δ (difference in lowest and highest percentages across racial/ethnic groups).

Figure 3 presents the percentages of racial/ethnic groups who reported consuming a subset of foods and beverages most commonly targeted under Prop 65. The greatest difference in the proportion of consumers of any single food category between any two racial/ethnic groups was observed for tamarind/chili candy—where zero consumers were reported among Caucasian and Middle Eastern participants, compared to 59.5% of Hispanics/Latinos. Similarly, stuffed grape leaves were consumed by neither Hispanic/Latinos nor Indian participants, compared to 38.9% of Middle Easterners.

Relative to all other groups, the proportion of nutritional powder consumers was 1.5 to 8.6-times higher among Caucasians, while the proportion of food paste consumers was 2.3 to 5.4-times higher among Indians, and the proportion of tostada/taco shell consumers and salsa consumers was 1.7 to 5.3-times higher and 1.5 to 3.6-times higher, respectively, among Hispanic participants. Chocolate beverage powder, tomato sauce, juice, and rice were among the foods where the proportions of consumers differed least between racial/ethnic groups.

**Figure 4:**
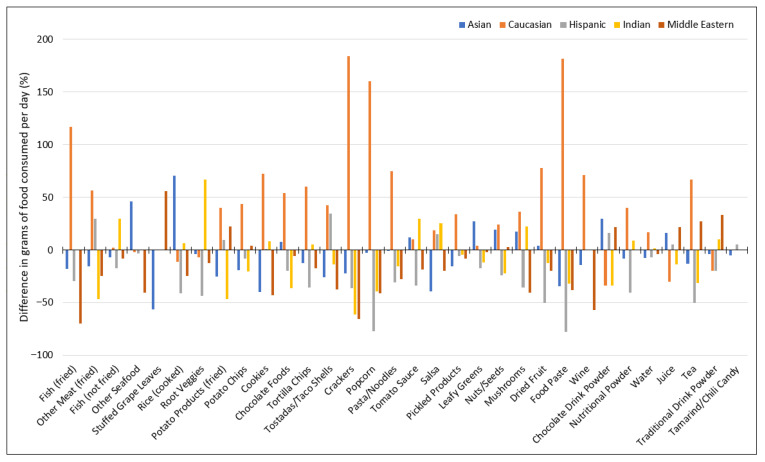
Percent difference in average daily intake (g/day) of foods and beverages by racial/ethnic groups relative to overall racial/ethnic Group Mean.

Figure 4 shows the percent difference in the average daily intake of foods and beverages by racial/ethnic group relative to the Group Mean across those racial/ethnic groups. Compared to the Group Mean within each food category, the daily intake of crackers, cookies, popcorn, pasta/noodles, dried fruit, food paste, wine, tea as well as fried fish and other fried meat was considerably (2.5- to 3.5-times) higher among Caucasians, while the daily intake of rice, root vegetables and grape leaves was 71%, 67% and 56% higher among Asians, Indians and Middle Easterners, respectively.

Table 3 summarizes results following multivariate regression analyses that included the covariates age (adults vs. teens) and race/ethnicity. Statistically significant differences in daily intake were observed for a similar set of food/beverage categories as those which showed the greatest percentage differences in Figure 4. Compared to Caucasians, for instance, daily intake of crackers, cookies, popcorn, pasta/noodles, and wine was statistically lower for most other racial/ethnic groups (*p* < 0.05). Similarly, the daily intakes of chocolate foods and nutritional powders were higher among Caucasians by up to 9.2 g and 17.0 g, respectively (*p* < 0.05). For Indian participants, root vegetable and total herb/spice daily intakes were statistically significantly higher than the Caucasian reference group by 65.5 g and 8.8 g, respectively, while for Hispanics tea and chili/tamarind candy intake differed significantly by −67.4 g and +3.7 g. Lastly, the average daily intake of rice was statistically higher (+59.1 g), and salsa (−10.3 g) and potato chips (−7.1 g) lower, among Asians relative to the reference group (*p* < 0.05).

### 3.5. Herb & Spice Intake

Table 4 presents the mean and median daily intakes and consumption frequencies of 20 herbs/spices per capita and among consumers-only. Herbs/spices consumed by the highest percentage (≥50%) of participants were garlic powder, basil, oregano, chili powder and paprika. Half of the herbs/spices categories were consumed at least once every other day by consumers, with masala mix being most frequently consumed (0.8 EO/day), followed by curry powder, paprika, garlic powder and chili powder (>0.6 EO/day) on average. Among participants consuming homegrown food, 46% reported none of their herbs/spices were grown at home.

Herbs/spices consumed in greatest daily abundance on average (per capita) were garlic powder (1.47 g/day), followed by chili powder (1.11 g/day), paprika (0.84 g/day), and turmeric (0.86 g/day). Masala mix was most abundantly consumed (2.26 g/day) when examining consumers only, followed by turmeric (2.23 g/day), chili powder (2.10 g/day) and garlic powder (1.95 g/day).

Mean and median herb/spice intake was 1.51 g/EO (interquartile range: 1.1–2.6 g/EO), with a mean and median volumetric intake of 0.83 tsp/EO and 0.75 tsp/EO, respectively, and mean and median mass intake of 1.2 g/day. When considering the herb/spice food category as a whole, the mean and median consumption frequency was 3.7 and 2.6 EO/day, and total mean and median daily mass intake was 8.3 and 2.7 g/day.

**Figure 5:**
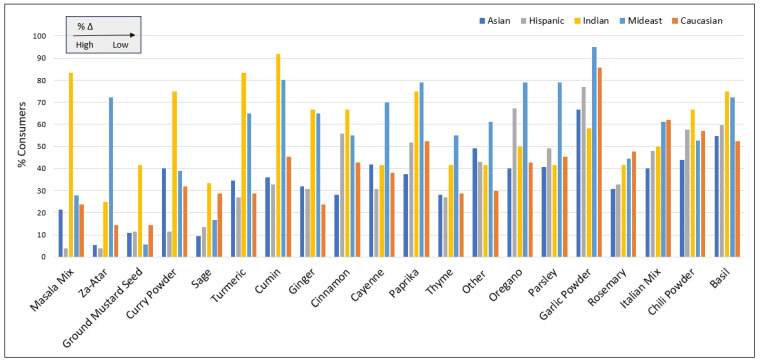
Percentages of racial/ethnic groups who reported consuming each herb/spice, ordered left-to-right based on %Δ (difference in lowest and highest percentages across racial/ethnic groups).

Figure 5 presents the percentages of racial/ethnic groups who reported consuming each herb/spice product, with the difference (Δ) between the highest and lowest percentage range within each product increasing from left to right. Masala mix showed the greatest Δ, with consumption reported by 83% of Indian participants, compared to just 4% among Hispanics. Similarly, 72.2% of Middle Eastern participants reported consuming za’atar, whereas no more than 25% of any other racial/ethnic group consumed this product. Consumption of cumin, turmeric and ginger was 2 to 3 times more prevalent among Indian and Middles Eastern participants compared to others. On average, roughly 60% of Indian and Middle Eastern participants reported consuming herbs/spices, compared to under 40% for all other groups.

Table 5 presents average consumption frequency (EO/day) of herbs/spices by racial/ethnic group, with statistical significance mostly observed among Indian participants, whose consumption frequency of 11 herbs/spices was significantly higher than the White/Caucasian reference group. Indian participants most frequently consumed masala, chili powder, cumin, turmeric and curry powder, each consumed over 5 times per week (>0.80 EO/day). Consumption frequency was also significantly higher among Middle Easterners for za’atar (0.34 EO/day) and parsley (0.58 EO/day) relative to the reference group (*p* < 0.01).

Masala mix showed the highest variance explained (r^2^ = 0.22) through multivariate analyses, followed by za’atar (r^2^ = 0.21), curry powder (r^2^ = 0.19), cumin (r^2^ = 0.19) and turmeric (r^2^ = 0.15). The overwhelming majority of teenage (>43%) and adult (>74%) participants reported to never rely on single serving size labels when consuming herbs/spices, with only 15% reporting to “often” or “always” rely on such labels (Chi-Square < 0.001).

**Figure 6:**
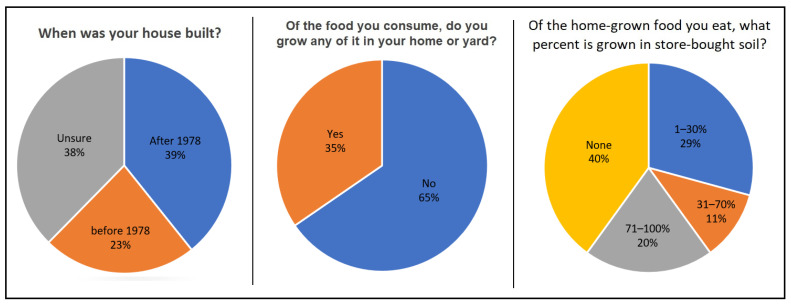
Distribution of survey responses regarding home construction year and gardening.

Figure 6 presents the response distribution of participants who answered questions about their home’s construction year, with 23.1% reporting a construction year < 1978, with 39.2% reporting later construction and 37.6% unsure. This distribution changed negligibly (±3%) when examining adults only. The proportion who reported growing at least some food at home was 35%. Of this proportion, 31% reported growing the majority of their homegrown food in store-bought soil (e.g., a raised garden bed), with 40% growing none of their food in store-bought soil.

## 4. Discussion

The primary contribution of this exploratory cross-sectional study is an examination of consumption patterns and differences by age and race/ethnicity of key staple foods and beverages, as well as those most commonly targeted under Prop 65. Several items (e.g., herbs/spices, nutritional powders) are excluded in many dietary intake studies and databases or are solely examined for their health benefits rather than their potential to expose people to contaminants through ingestion. Our results provide insights to adjust serving size recommendations and improve FFQ measurements to more realistically reflect consumption of these items overall and by race/ethnicity and age.

### 4.1. Food & Beverage Intake

Findings reveal the foods consumed in greatest daily abundance and by the highest percentage of survey respondents are those known to contain relatively high levels of heavy metals (e.g., root vegetables, rice, and leafy greens) compared to other foods [32,33,34,59]. These results have important health implications and underscore the need to develop protective exposure benchmarks that account for the impacts of multiple and cumulative exposures.

Stuffed grape leaves, though consumed by just 6% of participants, showed an average portion size (~6 stuffed leaves) far greater than the label-based serving sizes (~1 stuffed leaf) that typically accompany such products [56]. If contaminated, consuming an excess quantity of these products, as results indicate, would expose consumers to much higher levels of pollutants than would be predicted if using product labels to infer exposure. Further, among consumers, stuffed grape leaves and seafood were among the most abundantly consumed foods. Both may pose harm since stuffed grape leaves are typically stuffed with rice mixtures that contain heavy metals, while seafood is known to bioaccumulate environmental toxicants such as methylmercury and organic pollutants [35].

Consumption frequencies reported by participants tended to be roughly 2 times higher than those reported by the NHANES FFQ. This is expected given the expansive 1-year recall window included by NHANES, which is designed to maximize variability for use in statistical modeling and epidemiological research, not to estimate absolute food intake. Consequently, NHANES’s FFQ captures even those who rarely (once per year) eat certain foods, thus deflating average consumption frequencies [51]. When comparing mean portion sizes with 2-day NHANES data (as opposed to FFQ data) from 2017 to 2020 [60], results from this survey were 25% higher on average, potentially due to different age and racial/ethnic compositions and the limited sample size of our survey, while average daily intakes tended to be roughly 30% lower. This latter result is expected given the longer sampling window (1 week) of the current survey compared to NHANES (2 days), thus including less frequent consumers in our analysis.

The study also examined behavioral patterns that may affect food-related exposures, such as the repeated use of tea bags. Regarding beverages, tea was consumed in high volume and by ~half or more of participants. Roughly two-thirds of consumers said they used fresh teabags when drinking multiple cups of tea a day, suggesting the potential for greater lead leaching and exposure than what is conventionally assumed under Prop 65 [28].

Relative to adults, adolescents had greater portion sizes and daily intakes across nearly all foods examined in the survey, with statistically significant increases in daily intake (by roughly 50%) for rice, crackers, popcorn, tostadas/taco shells and juice. These findings are significant not only for potential heavy metal contamination, but also for acrylamide where foods are cooked at high temperatures [38,39,40]. With acrylamide being a carcinogen, this is especially relevant as young individuals are more vulnerable to cancer-causing agents. Moreover, adolescents likely weigh less than adults, resulting in potentially higher exposures per unit body mass.

This survey also revealed significant consumption differences by racial/ethnic group; e.g., while some racial/ethnic groups reported not consuming tamarind/chili candy, nearly two-thirds of Hispanic/Latinos reported eating such food. Similarly, over one-third of Middle Easterners were consumers of stuffed grape leaves compared to less than 5% of other groups. Other foods such as nutritional powder, rice, root vegetables, tostada/taco shells, salsa, and total herbs/spices also differed dramatically between racial/ethnic groups, although the variance explained by race/ethnicity ranged from moderate to low. These findings compliment prior studies that have reported dietary patterns to differ along racial/ethnic lines [1,2,47,61], signaling the importance of considering race/ethnic disparities when assessing food-related exposure or developing public health messages.

### 4.2. Herb/Spice Intake

Over half of herbs/spices included in this survey were consumed at least once every other day by consumers, with masala mix being the most frequently consumed on average, followed by curry powder, paprika, garlic powder and chili powder. Most of these herbs/spices showed average intakes of roughly 2 g/day or higher, with statistically significant increases in consumption frequency among Indian participants. Among Middle Easterners, an increased consumption frequency was observed for za’atar and parsley. Masala, za’atar, cumin, curry powder, and turmeric showed the highest variance explained by regression models.

For the herb/spice food category in aggregate, cumulative average daily intake was 8.3 g/day, and higher for certain racial/ethnic groups (e.g., Indians). Results suggest consumers typically ingest several different herbs/spices per day, in contrast to the assumptions that often underly Prop 65-related exposure assessments of herbs/spices, with vast differences in consumption by race/ethnicity. Cumulative intake exceeded studies that examined herb/spice consumption in Europe and America, yet was on par with research on Indian, Middle Eastern and Asian populations [45,46,47,48]. These findings help inform herb/spice-related chemical exposure assessment and showcase the critical underestimation of exposure (e.g., to lead) if herb/spice intake is assessed product-by-product instead of assessing total intake to the herb/spice food category as a whole. Such findings are particularly relevant given recent evidence identifying herbs/spices as the culprit behind elevated blood lead levels in Bangladeshi children living in New York City [62].

Compared to the FDA’s RACC industry guidance document, which identifies a standard portion size of ¼ teaspoon (or 0.5 g) for use on herb/spice nutritional labels, the average portion size reported in this study was over three-times higher. This suggests that RACC- and label-based estimates of portion sizes, if utilized in exposure assessment, will underestimate chemical exposures associated with such foods. The limited utility of using label-based serving sizes to inform herb/spice intake is supported by a complimentary finding from this survey which showed the overwhelming majority of respondents to “never” or “rarely” use label-based serving sizes when consuming herbs/spices. Moreover, that densities for products such as garlic powder and turmeric (≥3 g/tsp) vary so drastically from products such as sage and parsley (<1 g/tsp) [56], affirms the inappropriateness of a universal RACC metric for all herbs/spices particularly when conducting Prop-65-related exposure assessment.

### 4.3. Homegrown Food & Water Intake

Nearly a quarter of participants lived at a residence built before 1978 (when lead paint was banned in the U.S.), indicating potential lead exposure from their home (through ingested dust and fallen paint chips, especially among children) and surrounding environment (through indirect contamination of homegrown food). Moreover, one-third reported consuming food grown in their own yard and most did not utilize store-bought soil. These findings are significant given that lead paint is toxic and can flake off and contaminate residential soil, especially adjacent to a home structure, in turn contaminating the plants (and fruit) grown in such soils, particularly root vegetables (through direct absorption of lead from soil) and leafy greens (through direct absorption and dust accumulation). Use of store-bought soil, on the other hand, can help ensure cleaner growing conditions since some states have programs in place to ensure the safety of soils and fertilizers sold on the market [63]. In contrast, residential soils are usually not tested for soil contamination, which can render residents vulnerable. A recent analysis conducted in the study region (Santa Ana, CA, USA) demonstrated elevated carcinogenic and non-carcinogenic risk due to heavy metal contamination using over 1500 soil samples [64]. The study did not account for the prevalence of soil-to-food lead ingestion, which is a critical gap in the public health nutrition literature. Most participants ingested little-to-no daily water in the form of store-bought bottled water or water provided by a delivery service, and roughly a quarter of participants obtained most daily drinking water from unfiltered tap water. This suggests wide variability in public perceptions, preferences and/or access to water resources as well as different levels of vulnerability when considering water-related exposure to pollutants such as lead which can enter tap water through the post-treatment distribution system [65,66,67]. Importantly, the threat of lead exposure through unfiltered tap water consumption is also related to building age, as federal requirements to reduce the lead content of pipes, solder and plumbing fittings has become increasingly stringent over time (1986 onward) [68]. Thus, in the absence of home plumbing system updates and/or home water filtration, those living in older homes are expected to incur greater lead exposure through tap water ingestion.

### 4.4. Policy Implications

This study examined multiple aspects of food and beverage consumption relevant to California’s Prop 65 regulations. Rice was among the foods with the highest reported daily intake. While levels of contaminants such as lead are not often high in rice, under Prop 65 a California court in 2018 approved a consent judgment (which can inform future claims) that allows up to 0.056 ppm lead in rice without requiring a warning label [69]. Of greater influence, in 2023, a California trial court found a comparable amount of lead to be acceptable in rice grains [70]. That rice (as a food category) is frequently and abundantly consumed in the U.S., as affirmed by this study and others [71,72], suggests that rice is among the commodities most influential to population-wide lead exposure, and therefore important to strictly regulate. Additional regulatory review and action is therefore likely needed in California to protect consumers from lead exposure when they consume rice and other staple foods.

Single serving size values on food packaging, including those recommended in FDA’s RACC report, have become increasingly used to help estimate food intake and therefore food-related chemical exposure. This study, however, found some products (e.g., stuffed grape leaves, herbs/spices, etc.) to be consumed in far higher quantities, suggesting that label-based serving sizes can dramatically underestimate exposure. Such mischaracterization is not surprising given the wide variability of single serving sizes seen on products of the same food category (e.g., nearly identical packages of dried mushrooms often contain serving sizes that range 10-fold, from 5 g to 50 g) or the universal serving sizes on products that differ dramatically in density and use (e.g., FDA recommends all herb/spice labels use the same serving size value, 0.5 g). Combined with our finding that serving size labels are infrequently used, results suggest their limited utility for estimating food intake, underscoring population-based survey data as a better tool for assessing food intake—an approach which also closes the loophole through which companies might otherwise circumvent Prop 65 by merely modifying their serving size labels.

Further, under Prop 65, a court-approved consent judgment in 2021 deemed 0.018 ppm to be an allowable level of contamination for stuffed grape leaves. However, ingesting 242 g per serving (survey average) equates to an exposure of 4.3 µg in a day, exceeding the MADL by over 8-fold. Courts have also upheld 3 µg/day as an allowable level of lead in nutritional powders, and up to 4 µg/day when containing chocolate. This effectively establishes a similar exemption for lead contamination of up to 8 times the regulatory limit without the need to warn the public [73]. For chocolate products, a separate consent judgment allows 0.225 ppm lead and 0.960 cadmium, depending on cocoa content. Given such products were consumed by roughly 4 of 5 participants nearly every other day (based on this survey), such allowances place a large number of consumers unknowingly at risk.

When examining race/ethnicity, this study suggests that a failure to treat racial/ethnic groups separately when conducting exposure assessment may lead to disproportionate harm and environmental injustice. Similarly, where food preferences vary systematically by racial/ethnic group, product-specific pollution exemptions are akin to permits that allow companies to disproportionately pollute the environments of some, but not others. Previously noted consent judgements that allow lead in stuffed grape leaves and rice, for instance, place Asians and Middle Eastern consumers at disproportionate risk to harmful exposures, respectively. Such impacts are exacerbated by Prop 65’s limited complexity, which develops benchmarks for individual chemicals and exposures while ignoring the cumulative nature of real-world exposures that consumers experience throughout the day when ingesting multiple chemicals and foods. Studies by Hobe et al. (2023) and Hinojosa-Nogueira (2023) describe methods that improve upon such gaps [74,75]. 

Further, in some cases, pollution allowances under Prop 65 take the form of product-specific “naturally occurring” exemptions. An example is OEHHA’s recently adopted (2021) section under its Title 27 regulations [30], which allows up to 0.02 ppm lead in candies flavored with chili and/or tamarind. Our findings suggest that this statutory exemption disproportionately affects Hispanic/Latino consumers and adolescents. Other Prop 65 exemptions similarly concern foods consumed disproportionately by communities of color who already face systematic disadvantages and disproportionate exposures to environmental hazards like air, soil and water pollution in the U.S. [76,77,78,79].

While the Prop 65 statute considers such lead naturally occurring, research shows many such candies to contain lead below 0.02 ppm [30,80], suggesting that excessive lead contamination need not be accepted as inherent or “natural,” and can be largely avoided by supply chain modifications and/or changes in manufacturing, packaging or distribution processes. Similar findings apply to the pollution allowances for cocoa, grape leaves, rice and other product categories, in which many such foods show little contamination, thus demonstrating industry’s ability to comply with Prop 65. Based on our findings, Prop 65’s food category exemptions should be carefully reviewed and their pollution allowances sharply reduced and/or eliminated.

A primary contribution of this paper was its characterization of intake of various herbs/spices and estimation of cumulative intake across the herb/spice food category. With many herbs/spices show intake ≥2 g/day, and nearly 10 g/day cumulatively, this study demonstrates the underestimation of herb/spice intake that arises if ingestion of individual herbs/spices is treated separately under Prop 65. Under Prop 65, exposure assessment must be carried out across “general categories” (e.g., assessing total fish intake, rather than each species of fish), with the Prop 65 regulatory text invoking the USDA’s Home Economic Research Report [81] as the authority defining such categories. For the herb/spice food category, despite the USDA report explicitly establishing “seasonings, spices and herbs” as a single food category, courts and enforcement bodies have in some cases permitted industry to subdivide herbs/spices into separate food categories (e.g., basil, oregano, etc.) when conducting exposure assessment, resulting in potential exposure underestimation that allows harmful products to be sold without warning labels. The interchangeable nature and potentially overlapping use of herbs/spices as “flavor enhancers” make it essential to assess exposure to herbs/spices cumulatively, under a single food category, consistent with the USDA report and explicit Prop 65 requirement. 

### 4.5. Strengths and Limitations

An important strength of the study was the diverse sample which included adolescents and marginalized racial/ethnic groups. This was also a California-based survey that estimated daily intakes of foods and beverages targeted most commonly under Prop 65, thus having direct relevance to state policy. Moreover, this study examined the intake of herbs/spices and food powders, thus helping to advance exposure assessment of foods known to contain heavy metals and yet are not included in the 2-day NHANES database.

Limitations include the cross-sectional survey design which did not account for time-varying dietary patterns, including potential seasonal changes. Respondents were recruited using convenience sampling rather than a probability sampling approach, which resulted in the underrepresentation of White and Hispanic communities and overrepresentation of Asian communities relative to background statistics for Orange County. Summary findings for the sample population as a whole may therefore not be generalizable to the county or wider southern California region. Similarly, the sample sizes of our racial/ethnic analyses were, in some cases, low such that it may have compromised the generalizability to the wider population groups and lessened our ability to detect patterns and differences in dietary intake between groups. Also, since our regression models only adjusted for age and race/ethnicity, we cannot rule out the possibility of confounding by other socio-demographic terms.

While there is the possibility of selection bias due potentially to disproportionate participation by health-conscious people, who may have consumed foods more/less than average, we expect such bias to be minimal given that this survey was not marketed as a “health” survey. Given the reliance on self-reported data, there is also the possibility of social desirability bias, which may have resulted in some individuals underreporting their consumption of unhealthy foods (e.g., candies) while overreporting intake of healthy foods, though we expect such bias to be minimal given the anonymous, online nature of the survey.

Additionally, converting portion sizes to mass was carried out using standard volume-to-mass conversions that may not reflect the true quantity ingested by each consumer. However, this limitation is not expected to bias results with the exception of foods containing inedible components (e.g., meat with bones), which may have resulted in overestimation. Similarly, the conversion of consumption response ranges to discrete values may have introduced bias, though likely toward underestimating daily intake. Of critical importance, herb/spice intake may be drastically underestimated since many processed and/or prepared foods contain pre-added herbs/spices which are unknown to respondents and therefore unreported.

Finally, while this study characterized dietary intake, it did not estimate actual exposures to harmful contaminants. Similarly, it did not consider intentionally added contaminants regulated by laws beyond Prop 65, and did not consider the beneficial aspects of various foods (e.g., nutrient profiles), which may offset the deleterious impacts of some chemical exposures. For these reasons, this analysis lacked the exposure estimates required to weigh the pros and cons of consuming each food and to provide dietary guidance for consumers.

## 5. Conclusions

Findings identify root vegetables, rice, leafy greens, pasta/noodles, tea, juice, and seafood to be among the most heavily consumed foods by mass, which is relevant to exposure assessment since many such foods contain elevated heavy metals. Results also showed daily intake of specific foods such as stuffed grape leaves, tamarind/chili candy and herbs/spices to vary by age and race/ethnicity, suggesting that many of Prop 65’s pollution allowances are likely exacerbating issues of health inequity and environmental injustice. Moreover, findings from this study indicate that the methods of exposure assessment often applied under Prop 65, especially relating to herbs/spices, are likely to underestimate single-day exposures, thus allowing unsafe products on the market without warning labels. Study outcomes are broadly relevant to environmental health and nutrition science, with particular relevance to public health practitioners and California’s Prop 65 regulators and other stakeholders.

## Figures and Tables

**Table 1:** Characteristics of sample population (N = 186).

Race/Ethnicity	**%**	N
Asian/Pacific Islander	40.9	76
Latino/Hispanic	28.0	52
White/Caucasian	11.8	22
Middle Eastern	11.8	22
Indian	6.5	12
Other	1.1	2
Age		
13–17	39.2	74
18–24	46.6	88
25–39	10.1	19
40–59	4.2	8
Gender		
Male	36.0	68
Female	62.9	119
Other	1.1	2
Education		
No High School Diploma	39.9	75
High School Diploma	20.2	38
Some College	23.4	44
Bachelor’s Degree	6.9	13
Graduate/Professional Degree	9.5	18
Annual Household Income		
<$25 K	21.5	40
$25–50 K	14.5	27
$50–100 K	24.7	46
$100–200 K	29.6	55
>$200 K	9.7	18

**Table 2:** Mean and median intake (g/person/day), along with other summary statistics for select foods and beverages included in dietary survey.

	All Participants N = 186				Among Consumers Only			
	N	(%)	Mean Daily Intake (g)	Median Daily Intake (g)	Mean Portion Size (g)	Mean Consumption Frequency (EO/day)	Mean Daily Intake (g)	Median Daily Intake (g)
Fruits/Vegetables								
Root Vegetables	182	96.3	83.9	48.3	128.6	0.55	87.2	48.3
Leafy Greens	159	84.1	33.6	29.4	64.3	0.52	39.5	29.4
Potato Products (fried)	136	71.4	29.8	18.9	126.9	0.32	41.7	27.7
Dried Fruit	58	30.7	6.8	0.0	59.2	0.41	22.2	15.2
Grains								
Rice (cooked)	172	91.0	52.6	37.2	82.9	0.60	57.8	37.2
Pasta/Noodles	153	81.0	30.9	22.5	108.2	0.33	38.0	33.8
Tostadas/Taco Shells	56	29.6	1.3	0.0	17.9	0.21	4.2	2.6
Meat								
Fish (fried)	39	20.6	4.2	0.0	95.4	0.19	20.3	14.2
Fish (not fried)	87	46.0	11.1	0.0	90.1	0.28	24.1	14.2
Other Seafood	74	39.2	21.8	0.0	211.9	0.28	55.8	48.5
Other Meat (fried)	107	56.6	20.2	6.1	119.1	0.28	35.7	20.2
Sauces/Pastes								
Tomato Sauce	125	65.6	16.9	11.6	92.0	0.27	25.8	11.6
Salsa	84	44.4	10.4	0.0	54.1	0.34	23.2	12.6
Food Paste	52	27.5	4.2	0.0	29.6	0.39	15.0	9.7
Snacks								
Cookies	124	65.6	15.3	5.7	58.5	0.33	23.3	13.4
Crackers	74	39.2	7.7	0.0	53.2	0.31	19.7	8.5
Potato Chips	120	63.5	8.8	5.4	38.1	0.34	13.8	7.5
Tortilla Chips	87	46.0	5.1	0.0	37.7	0.29	11.1	6.4
Nuts/Seeds	112	59.3	14.9	4.9	48.3	0.49	25.0	19.4
Chocolate Foods	149	78.8	9.6	6.1	30.0	0.42	12.2	6.1
Pickled Products	85	45.0	10.1	0.0	64.8	0.33	22.4	14.2
Popcorn	64	21.7	1.2	0.0	17.8	0.31	5.4	1.1
Tamarind/Chili Candy	33	24.1 ^c^	1.5	0.0	15.9	0.34	6.4	3.0
Beverages								
Water	186	100	1413	1440	240 ^a^	1.0	1436	1440
Juice	113	59.8	72.4	68.6	240 ^a^	0.42	120.5	68.6
Tea	87	46.0	70.2	0.0	240 ^a^	0.65	151.7	68.6
Wine	14	7.4	7.9	0.0	150 ^a^	0.47	105.7	51.9
Food/Beverage Powders								
Nutritional Powder	52	27.5	6.8	0.0	44.3 ^b^	0.58	24.3	12.7
Traditional Drink Powder	63	33.3	0.3	0.0	2.1 ^b^	0.36	0.8	0.6
Chocolate Drink Powder	42	22.2	2.4	0.0	23.3 ^b^	0.49	10.8	6.7
Other								
Mushrooms	69	36.5	8.2	0.0	34.4	0.31	22.5	14.8
Stuffed Grape Leaves	11	5.8	2.6	0.0	242.2	0.19	44.5	19.3

^a^ Assumed to be 1 cup (8 oz) for non-alcoholic liquids, and 1 glass (5 oz) for wine (1 oz = 30 g). ^b^ Value based on USDA FoodData Central database. ^c^ Percentage adjusted to account for missing survey responses.

**Table 3:** Effect estimates of daily intake (g/day) of foods and beverages following multivariate regression analyses.

				Race/ethnicity			Age Category
	r^2^	Int.	Asian	Hispanic	Indian	Middle Eastern	Adult
**Beverages**							
Juice	0.06	**58.17**	**38.74**	**46.28**	22.63	**54.10**	**−36.08**
Tea	0.10	**81.93**	−36.82	**−67.35**	−35.99	19.55	**33.76**
Wine	0.05	**29.68**	−23.91	**−26.66**	**−29.68**	**−23.91**	0.00
**Fruits/Veggies**							
Root Veggies	0.08	**92.26**	6.21	−34.26	**65.59**	9.09	−24.34
Potato Products (fried)	0.05	**38.85**	**−13.70**	−2.44	−17.25	2.09	−1.42
Dried Fruit	0.07	**17.02**	**−11.08**	**−14.58**	−8.51	**−15.08**	1.16
**Grains**							
Rice (cooked)	0.27	**50.93**	**59.05**	−4.16	20.19	23.79	**−41.38**
Pasta/Noodles	0.07	**52.27**	**−15.19**	**−29.10**	−20.29	**−23.86**	−4.44
Tostadas/Taco Shells	0.18	**2.24**	**−1.12**	0.89	−1.14	−1.11	**−0.89**
**Meat**							
Fish (fried)	0.03	**7.95**	−4.68	**−5.90**	**−0.11**	**−7.25**	0.57
**Sauces/Pastes**							
Tomato Sauce	0.03						
Salsa	0.18	**17.42**	**−10.32**	6.15	−8.74	−7.48	−4.67
Food Paste	0.03	**9.32**	−4.84	**−8.00**	2.66	−3.84	−0.95
**Snacks**							
Cookies	0.08	**27.67**	**−18.45**	**−12.06**	**−8.58**	−17.03	−2.22
Crackers	0.10	**8.48**	**−4.29**	**−5.26**	**−6.06**	**−5.52**	**−2.56**
Potato Chips	0.07	**15.83**	**−7.11**	−3.82	−6.69	**−6.47**	−2.88
Tortilla Chips	0.07	**12.04**	**−6.36**	**−5.83**	**−6.48**	**−6.21**	−2.53
Chocolate Foods	0.06	**15.01**	**−4.35**	**−8.63**	**−9.18**	**−7.02**	0.52
Popcorn	0.13	**5.43**	**−3.16**	**−4.41**	**−3.58**	**−2.77**	**−1.89**
Tamarind/Chili Candy	0.13	0.09	0.70	**3.71**	0.00	0.12	−0.21
**Food/Beverage Powders**							
Nutritional Powder	0.12	**17.04**	**−12.27**	**−13.72**	−8.01	**−18.16**	1.83
Traditional Drink Powder	0.05	0.10	**0.38**	0.16	**0.61**	**0.46**	0.14
Chocolate Drink Powder	0.01	**0.53**	**−0.15**	**−0.30**	**−0.32**	**−0.25**	0.02
Total Herbs/Spices	0.08	**12.3**	−1.8	−7.0	**13.1**	3.3	−4.6
**Other**							
Stuffed Grape Leaves	0.18	1.96	−2.08	−2.47	−2.47	**18.44**	1.15

Note 1: “Int.” indicates intercept. Note 2: Statistical significance indicated by bold (*p* < 0.01) and underlined (*p* < 0.05) font. Note 3: The reference group for race/ethnicity was “Caucasian” and for age was “Adolescents”.

**Table 4:** Mean and median intake (g/person/day) of herbs/spices evaluated from dietary survey, along with other summary statistics, listed in descending order of percent consumers.

	All Participants N = 186						Consumers Only	
	N	(%)	Mean Daily Intake (g)	Median Daily Intake (g)	Unit Conversion (g/tsp) ^a^	Mean Consumption Frequency (EO/day)	Mean Daily Intake (g)	Median Daily Intake (g)
Garlic Powder	139	75.1	1.47	0.66	3.1	0.61	1.95	0.66
Basil	108	59.0	0.28	0.08	1.1	0.43	0.47	0.24
Oregano	98	53.3	0.31	0.10	1.4	0.42	0.58	0.30
Chili Powder	97	52.7	1.11	0.19	2.7	0.61	2.10	1.16
Paprika	92	50.0	0.84	0.08	2.3	0.62	1.68	0.49
Italian Mix	88	48.1	0.34	0	1.8 ^b^	0.42	0.71	0.39
Parsley	87	47.5	0.12	0	0.5	0.49	0.26	0.11
Other	80	45.7	0.67	0	1.8 ^b^	0.74	1.46	0.84
Cumin	84	45.2	0.54	0	1.8 ^b^	0.55	1.19	0.77
Cinnamon	81	43.8	0.58	0	2.6	0.47	1.34	0.56
Cayenne	77	41.8	0.43	0	1.8 ^b^	0.45	1.05	0.39
Turmeric	72	38.9	0.86	0	3.0	0.59	2.23	0.64
Ginger	68	36.8	0.39	0	1.8	0.51	1.06	0.39
Rosemary	63	34.4	0.21	0	1.2	0.42	0.62	0.26
Curry Powder	62	33.7	0.56	0	2.0	0.63	1.66	0.86
Thyme	60	32.4	0.19	0	1.2	0.40	0.59	0.26
Masala Mix	39	21.3	0.48	0	1.8 ^b^	0.80	2.26	0.96
Sage	28	15.3	0.06	0	0.7	0.46	0.40	0.15
Za-Atar	25	13.7	0.17	0	1.8 ^b^	0.53	1.24	0.45
Ground Mustard Seed	24	13.3	0.19	0	2.0	0.56	1.41	0.64
Average			0.49		1.8	0.54	1.21	0.53

**Table 5:** Effect estimates of consumption frequency of herbs/spices following multivariate regression analyses.

	R^2^	Int.	Asian	Hispanic	Indian	Middle Eastern	Caucasian	Adult	Teenage
Garlic Powder	0.03	0.51	−0.16	−0.05	−0.23	−0.02	0.00	0.05	0.00
Basil	0.08	0.25	0.02	0.00	**0.38**	0.14	0.00	−0.08	0.00
Oregano	0.05	0.26	−0.06	0.03	0.07	0.16	0.00	−0.07	0.00
Chili Powder	0.10	0.42	−0.12	−0.16	**0.49**	−0.19	0.00	−0.03	0.00
Paprika	0.09	0.33	−0.12	−0.10	**0.47**	0.17	0.00	0.01	0.00
Italian Mix	0.04	0.27	**−0.13**	−0.07	−0.06	−0.08	0.00	0.03	0.00
Parsley	0.14	0.26	−0.01	0.04	0.11	**0.32**	0.00	**−0.13**	0.00
Other	0.07	0.28	0.18	0.10	**0.41**	**0.47**	0.00	**−0.22**	0.00
Cumin	0.19	0.14	0.02	−0.01	**0.76**	0.18	0.00	0.06	0.00
Cinnamon	0.05	0.26	**−0.16**	−0.07	0.12	−0.08	0.00	0.05	0.00
Cayenne	0.05	0.14	0.08	−0.03	**0.20**	0.14	0.00	−0.02	0.00
Turmeric	0.15	0.13	0.04	−0.05	**0.75**	0.12	0.00	0.04	0.00
Ginger	0.09	0.08	0.10	0.02	**0.36**	**0.19**	0.00	0.02	0.00
Rosemary	0.09	0.23	0.00	−0.02	0.03	0.09	0.00	−**0.14**	0.00
Curry Powder	0.19	0.16	0.05	−0.11	**0.70**	−0.04	0.00	0.02	0.00
Thyme	0.06	0.11	0.03	0.03	0.21	0.12	0.00	−0.05	0.00
Masala Mix	0.22	0.16	0.00	−0.13	**0.84**	0.00	0.00	−0.02	0.00
Sage	0.02	0.10	−0.04	0.00	0.10	0.01	0.00	−0.04	0.00
Za-Atar	0.21	0.08	−0.03	−0.06	0.07	**0.26**	0.00	−0.01	0.00
Ground Mustard Seed	0.06	0.04	0.01	−0.02	**0.24**	−0.06	0.00	0.03	0.00

Note 1: “Int.” indicates intercept. Note 2: Statistical significance indicated by bold (*p* < 0.01) and underlined (*p* < 0.05) font. Note 3: The reference group for race/ethnicity was “Caucasian” and for age was “Adolescents”.

## Data Availability

The raw data supporting the conclusions of this article will be made available by the authors on request.

## Supplementary Materials

The following supporting information can be downloaded at: https://www.mdpi.com/article/10.3390/nu17193149/s1, Table S1. List of all survey questions relating to food/beverage consumption, homegrown food, and housing age.

## References

[1] Kell K.P., Judd S.E., Pearson K.E., Shikany J.M., Fernández J.R. (2015). Associations between socio-economic status and dietary patterns in US black and white adults. Br. J. Nutr..

[2] Chen L., Zhu H., Gutin B., Dong Y. (2019). Race, Gender, Family Structure, Socioeconomic Status, Dietary Patterns, and Cardiovascular Health in Adolescents. Curr. Dev. Nutr..

[3] U.S. Food and Drug Administration (2025). Chemical, Metals, Natural Toxins & Pesticides Guidance Documents & Regulations. Several Foods and Beverages are Known to Disproportionately Accumulate Contaminants.

[4] European Union (2024). Commission Regulation (EU) 2024/1756 of 25 June 2024 Amending and Correcting Regulation (EU) 2023/915 on Maximum Levels for Certain Contaminants in Food. https://eur-lex.europa.eu/eli/reg/2024/1756/oj.

[5] World Health Organization (2017). Guidelines for Drinking Water: Fourth Edition Incorporating the First Addendum. https://iris.who.int/bitstream/handle/10665/254637/9789241549950-eng.pdf?sequence=1&isAllowed=y#page=365.

[6] California Office of Environmental Health Hazard Assessment (OEHHA) (2023). Proposition 65 List Of Carcinogens or Reproductive Toxicants. https://oehha.ca.gov/media/downloads/proposition-65//p65chemicalslist.pdf.

[7] California Legislative Information (2007). 1. CHAPTER 6.6. Safe Drinking Water and Toxic Enforcement Act of 1986: CA Health and Safety Code, Section 25249.5.

[8] OEHHA Proposition 65 in Plain Language|OEHHA. Ca.Gov 2013. https://oehha.ca.gov/proposition-65/general-info/proposition-65-plain-language.

[9] Office of Environmental Health Hazard Assessment (2019). Proposition 65 No Significant Risk Levels (NSRLs) for Carcinogens and Maximum Allowable Dose Levels (MADLs) for Chemicals Causing Reproductive Toxicity. https://www.p65warnings.ca.gov/chemicals.

[10] Wu K.G., Chang C.Y., Yen C.Y., Lai C.C. (2019). Associations between environmental heavy metal exposure and childhood asthma: A population-based study. J. Microbiol. Immunol. Infect..

[11] Boskabady M., Marefati N., Farkhondeh T., Shakeri F., Farshbaf A., Boskabady M.H. (2018). The effect of environmental lead exposure on human health and the contribution of inflammatory mechanisms, a review. Environ. Int..

[12] Wang I.J., Karmaus W.J., Yang C.C. (2017). Lead exposure, IgE, and the risk of asthma in children. J. Expo. Sci. Environ. Epidemiol..

[13] Pugh Smith P., Nriagu J.O. (2011). Lead poisoning and asthma among low-income and African American children in Saginaw, Michigan. Environ. Res..

[14] Grandjean P., Landrigan P.J. (2014). Neurobehavioural effects of developmental toxicity. Lancet Neurol..

[15] Reuben A., Caspi A., Belsky D.W., Broadbent J., Harrington H., Sugden K., Houts R.M., Ramrakha S., Poulton R., Moffitt T.E. (2017). Association of childhood blood lead levels with cognitive function and socioeconomic status at age 38 years and with IQ change and socioeconomic mobility between childhood and adulthood. JAMA J. Am. Med. Assoc..

[16] Canfield R.L., Henderson C.R., Cory-Slechta D.A., Cox C., Jusko T.A., Lanphear B.P. (2003). Intellectual Impairment in Children with Blood Lead Concentrations below 10 ug per Deciliter. N. Engl. J. Med..

[17] Lanphear B.P., Hornung R., Khoury J., Yolton K., Baghurst P., Bellinger D.C., Canfield R.L., Dietrich K.N., Bornschein R., Greene T. (2005). Low-level environmental lead exposure and children’s intellectual function: An international pooled analysis. Environ. Health Perspect..

[18] Kennedy D.A., Woodland C., Koren G. (2012). Lead exposure, gestational hypertension and pre-eclampsia: A systematic review of cause and effect. J. Obstet. Gynaecol..

[19] Poropat A.E., Laidlaw M.A.S., Lanphear B., Ball A., Mielke H.W. (2018). Blood lead and preeclampsia: A meta-analysis and review of implications. Environ. Res..

[20] Taylor C.M., Golding J., Emond A.M. (2015). Adverse effects of maternal lead levels on birth outcomes in the ALSPAC study: A prospective birth cohort study. BJOG An Int. J. Obstet. Gynaecol..

[21] Xie X., Ding G., Cui C., Chen L., Gao Y., Zhou Y., Shi R., Tian Y. (2013). The effects of low-level prenatal lead exposure on birth outcomes. Environ. Pollut..

[22] Wu A.H., Wu J., Tseng C., Yang J., Shariff-Marco S., Fruin S., Larson T., Setiawan V.W., Masri S., Porcel J. (2020). Association Between Outdoor Air Pollution and Risk of Malignant and Benign Brain Tumors: The Multiethnic Cohort Study. JNCI Cancer Spectr..

[23] Nduka J.K., Kelle H.I., Amuka J.O. (2019). Health risk assessment of cadmium, chromium and nickel from car paint dust from used automobiles at auto-panel workshops in Nigeria. Toxicol. Reports.

[24] Järup L., Berglund M., Elinder C.G., Nordberg G., Vahter M. (1998). Health effects of cadmium exposure—A review of the literature and a risk estimate. Scand. J. Work. Environ. Health.

[25] Anetor J.I., Wanibuchi H., Fukushima S. (2007). Arsenic exposure and its health effects and risk of cancer in developing countries: Micronutrients as host defence. Asian Pacific J. Cancer Prev..

[26] Hutton M., Hutchinson T.C., Meema K. (1987). Human Health Concerns of Lead, Mercury, Cadmium and Arsenic. Lead, Mercury, Cadmium and Arsenic in the Environment.

[27] Jang Y.-C., Somanna Y., Kim H. (2016). Source, Distribution, Toxicity and Remediation of Arsenic in the Environment—A review. Int. J. Appl. Environ. Sci..

[28] Superior Court of the State of California County of San Francisco (2017). Whitney R. Leeman v. Starbucks Corporation.

[29] Lynch R.A., Boatright D.T., Moss S.K. (2000). Lead-contaminated imported tamarind candy and children’s blood lead levels. Public Health Rep..

[30] California Environmental Protection Agency (2021). Title 27. Environmental Protection Division 4. Office Of Environmental Health Hazard Assessment Chapter 3. Naturally Occurring Lead In Candy § 28500. Naturally Occurring Levels of Lead In Candy.

[31] State of California Department of Justice (2024). Annual Reports of Settlements. https://oag.ca.gov/prop65/annual-settlement-reports.

[32] Sultana R., Tanvir R.U., Hussain K.A., Chamon A.S., Mondol M.N. (2022). Heavy Metals in Commonly Consumed Root and Leafy Vegetables in Dhaka City, Bangladesh, and Assessment of Associated Public Health Risks. Environ. Syst. Res..

[33] Singh S. (2012). Heavy metals accumulation and distribution pattern in different vegetable crops. J. Environ. Chem. Ecotoxicol..

[34] Zwolak A., Sarzyńska M., Szpyrka E., Stawarczyk K. (2019). Sources of Soil Pollution by Heavy Metals and Their Accumulation in Vegetables: A Review. Water Air Soil Pollut..

[35] Chen C.Y., Serrell N., Evers D.C., Fleishman B.J., Lambert K.F., Weiss J., Mason R.P., Bank M.S. (2008). Meeting report: Methylmercury in marine ecosystems--from sources to seafood consumers. Environ. Health Perspect..

[36] Braun J.M., Hauser R. (2011). Bisphenol A and children’s health. Curr. Opin. Pediatr..

[37] Zheng J., Tian L., Bayen S. (2023). Chemical contaminants in canned food and can-packaged food: A review. Crit. Rev. Food Sci. Nutr..

[38] Virk-Baker M.K., Nagy T.R., Barnes S., Groopman J. (2014). Dietary acrylamide and human cancer: A systematic review of literature. Nutr. Cancer.

[39] Lineback D.R., Coughlin J.R., Stadler R.H. (2012). Acrylamide in foods: A review of the science and future considerations. Annu. Rev. Food Sci. Technol..

[40] Office of Environmental Health Hazard Assessment (OEHHA) (2022). § 25505. Exposures to Listed Chemicals in Cooked or Heat Processed Foods.

[41] U.S. Food and Drug Administration (2022). Draft Guidance for Industry: Action Levels for Lead in Juice. https://www.fda.gov/regulatory-information/search-fda-guidance-documents/draft-guidance-industry-action-levels-lead-juice.

[42] Shen F.M., Chen H.W. (2008). Element composition of tea leaves and tea infusions and its impact on health. Bull. Environ. Contam. Toxicol..

[43] Han W.Y., Zhao F.J., Shi Y.Z., Ma L.F., Ruan J.Y. (2006). Scale and causes of lead contamination in Chinese tea. Environ. Pollut..

[44] Mackonochie M., Rodriguez-Mateos A., Mills S., Rolfe V. (2023). A Scoping Review of the Clinical Evidence for the Health Benefits of Culinary Doses of Herbs and Spices for the Prevention and Treatment of Metabolic Syndrome. Nutrients.

[45] Karam L., Kosseifi N., Jaoude M.A., Merhi S., Elobeid T., Hassan H.F. (2022). The influence of socio-demographic factors on patterns of thyme and thyme products consumption: The case of a Mediterranean country. Food Sci. Technol..

[46] Sirguri V., Bhat R. (2015). Assessing intake of spices by pattern of spice use, frequency of consumption and portion size ofspices consumed from routinely prepared dishesin southern India.pdf. Nutr. J..

[47] Bhathal S.K., Kaur H., Bains K., Mahal A.K. (2020). Assessing intake and consumption level of spices among urban and rural households of Ludhiana district of Punjab, India. Nutr. J..

[48] Carlsen M.H., Blomhoff R., Andersen L.F. (2011). Intakes of culinary herbs and spices from a food frequency questionnaire evaluated against 28-days estimated records. Nutr. J..

[49] Guasch-Ferré M., Willett W.C. (2021). The Mediterranean diet and health: A comprehensive overview. J. Intern. Med..

[50] United States Centers for Disease Control and Prevention: National Center for Health Statistics (2025). NHANES Questionnaires, Datasets, and Related Documentation. https://wwwn.cdc.gov/nchs/nhanes/Default.aspx.

[51] National Center for Health Statistics (2004). National Health and Nutrition Examination Survey (NHANES): 2003–2004 Data Documentation, Codebook, and Frequencies.

[52] State of California Department of Justice 60-Day Notice Search. https://oag.ca.gov/prop65/60-day-notice-search.

[53] United States Census Bureau California: 2020 Census. https://www.census.gov/library/stories/state-by-state/california-population-change-between-census-decade.html.

[54] National Cancer Institute Division of Cancer Control & Population Sciences Usual Dietary Intakes: NHANES Food Frequency Questionnaire (FFQ). https://epi.grants.cancer.gov/diet/usualintakes/ffq.html.

[55] National Institutes of Health (2018). Diet History Questionnaire.

[56] United States Department of Agricuture (USDA) (2024). USDA FoodData Central. https://fdc.nal.usda.gov/.

[57] U.S. Food and Drug Administration (2018). Reference Amounts Customarily Consumed: List of Products for Each Product Category: Guidance for Industry. http://www.fda.gov/FoodGuidances.

[58] SAS Institute Inc (2014). SAS® 9.4 Statements: Reference.

[59] Gilbert-Diamond D., Cottingham K.L., Gruber J.F., Punshon T., Sayarath V., Gandolfi A.J., Baker E.R., Jackson B.P., Folt C.L., Karagas M.R. (2011). Rice consumption contributes to arsenic exposure in US women. Proc. Natl. Acad. Sci. USA.

[60] Food Surveys Research Group. Agricultural Research Service. United States Department of Agriculture What We Eat in America: Documentation and Data Sets. https://www.ars.usda.gov/northeast-area/beltsville-md-bhnrc/beltsville-human-nutrition-research-center/food-surveys-research-group/docs/wweia-documentation-and-data-sets/.

[61] Lin S., Herdt-Losavio M.L., Chen M., Luo M., Tang J., Hwang S.A. (2014). Fish consumption patterns, knowledge and potential exposure to mercury by race. Int. J. Environ. Health Res..

[62] Emanuel G. (2024). You’ll never guess the culprit in a global lead poisoning mystery. National Public Radio: All Things Considered.

[63] California Department of Food and Agriculture (2025). Fertilizing Materials Inspection Program. https://www.cdfa.ca.gov/is/ffldrs/fertilizer.html.

[64] Masri S., LeBrón A., Logue M., Valencia E., Ruiz A., Reyes A., Lawrence J.M., Wu J. (2020). Social and spatial distribution of soil lead concentrations in the City of Santa Ana, California: Implications for health inequities. Sci. Total Environ..

[65] Roy S., Edwards M.A. (2019). Preventing another lead (Pb) in drinking water crisis: Lessons from the Washington D.C. and Flint MI contamination events. Curr. Opin. Environ. Sci. Health.

[66] Boyd G.R., Pierson G.L., Kirmeyer G.J., English R.J. (2008). Lead variability testing in Seattle Public Schools. J. Am. Water Work. Assoc..

[67] Murphy E.A. (1993). Effectiveness of Flushing on Reducing Lead and Copper Levels in School Drinking Water. Environ. Health Perspect..

[68] United States Environmental Protection Agency (USEPA) (2025). Use of Lead Free Pipes, Fittings, Fixtures, Solder, and Flux for Drinking Water. Safe Drinking Water Act. https://www.epa.gov/sdwa/use-lead-free-pipes-fittings-fixtures-solder-and-flux-drinking-water.

[69] Superior Court of California County of Los Angeles (2018). Settlement Agreement Between Plaintiffs California Rice Commission and USA Rice Federation, and Defendant Consumer Advocacy Group, Inc..

[70] Superior Court of California County of Los Angeles (2023). Consumer Advocacy Group, Inc. v Gulf Pacific Rice Co: Lead Case No. BC553427.

[71] Childs N. (1993). Nathan Childs Americans Are Eating More Rice. Res. Agric. Appl. Econ..

[72] Batres-Marquez S.P., Jensen H.H., Upton J. (2009). Rice Consumption in the United States: Recent Evidence from Food Consumption Surveys. J. Am. Diet. Assoc..

[73] Superior Court of the State of California County of Fresno (2019). Environmental Law Foundation v. Protein Supplements.

[74] Hinojosa-Nogueira D., Muros J.J., Navajas-Porras B., Delgado-Osorio A., Pérez-Burillo S., Pastoriza S., Rufián-Henares J. (2023). Development of a food composition database of different food contaminants CONT11 and estimation of dietary exposure in children of southern Spain. Food Chem. Toxicol..

[75] Hobé R.G., Van Asselt E.D., Van Den Heuvel L., Hoek-van Den Hil E.F., Van Der Fels-Klerx H.J. (2023). Methodology for risk-based monitoring of contaminants in food—A case study in cereals and fish. Food Res. Int..

[76] Mikati I., Benson A.F., Luben T.J., Sacks J.D., Richmond-Bryant J. (2018). Disparities in distribution of particulate matter emission sources by race and poverty status. Am. J. Public Health.

[77] Gaffron P., Niemeier D. (2015). School locations and traffic Emissions—Environmental (In)justice findings using a new screening method. Int. J. Environ. Res. Public Health.

[78] United Church of Christ Commission for Racial Justice (1987). Toxic Waste and Race in the United States: A National Report on the Racial and Socio-Economic Characteristics of Communities with Hazardous Waste Sites.

[79] Rosofsky A., Levy J.I., Zanobetti A., Janulewicz P., Fabian M.P. (2018). Temporal trends in air pollution exposure inequality in Massachusetts. Environ. Res..

[80] Tamayo-Ortiz M., Sanders A.P., Rosa M.J., Wright R.O., Amarasiriwardena C., Mercado-García A., Pantic I., Lamadrid-Figueroa H., Téllez-Rojo M.M. (2020). Lead concentrations in Mexican candy: A follow-up report. Ann. Glob. Health.

[81] Pao E.M., Fleming K.H., Guenther P.M., Mickle S.J. (1982). Foods Commonly Eaten by Individuals: Amount Per Day and Per Eating Occasion.

