# Supplementary material for: Assessing Dietary Consumption of Toxicant-Laden Foods and Beverages by Age and Ethnicity in California: Implications for Proposition 65
Source: Nutrients. 2025 Oct 2;17(19):3149. doi: 10.3390/nu17193149 (PMC12525620; doi:10.3390/nu17193149)
Supplement: Supplementary file 1 [file nutrients-17-03149-s001.zip › nutrients-3872422-supplementary-final update.pdf]

Supplemental Materials

Table S1. List of all survey questions relating to food/beverage consumption, homegrown food, and housing age.

When was your residence built?
In the past 7 days, how often did you eat battered fried fish (e.g., fish sticks, in a fish taco, etc.)?
Each time you ate battered fried fish, how much did you usually eat?
In the past 7 days, how often did you eat other fried meat that has been breaded and/or battered (not including fish)? (i.e., fried chicken, fried shrimp, fried calamari, etc.)
Each time you ate other fried meat that has been breaded and/or battered, how much did you usually eat?
In the past 7 days, how often did you eat fish, other than fish that was battered and/or fried?
Each time you ate non-fried, non-battered fish, how much did you usually eat?
In the past 7 days, how often did you eat seafood other than fish (e.g., lobster, crab, shrimp, scallop, mussels, squid, octopus, etc.)?
Each time you ate seafood other than fish (e.g., lobster, crab, shrimp, scallop, mussels, squid, octopus, etc.), how much did you usually eat?
In the past 7 days, how often did you eat stuffed grape leaves?
Each time you ate stuffed grape leaves, how many did you usually eat?
In the past 7 days, how often did you eat cooked rice?
Each time you ate rice, how much did you usually eat? (Assume rice is cooked)
In the past 7 days, how often did you eat root vegetables (e.g., potatoes, carrots, radishes, onions, garlic, etc.)?
Each time you ate root vegetables (e.g., potatoes, carrots, radishes, onions, garlic, etc.), how much did you usually eat? (Assume 1 cup = 1 medium-sized potato)
In the past 7 days, how often did you eat fried potatoes (e.g., French fries, tater tots, hash browns, etc.)?

Each time you ate fried potatoes (e.g., French fries, tater tots, hash browns, etc.), how much did you usually eat?
In the past 7 days, how often did you eat potato chips?
Each time you ate potato chips, how much did you usually eat?
In the past 7 days, how often did you eat cookies?
Each time you ate cookies, how many did you usually eat?
In the past 7 days, how often did you eat products (excluding beverages) composed primarily of chocolate? (e.g., chocolate bar, fudge brownie, etc.)
Each time you ate products (excluding beverages) primarily composed of chocolate (e.g., chocolate bar, fudge brownie, etc.), how much did you usually eat?
In the past 7 days, how often did you eat tortilla chips?
Each time you ate tortilla chips, how much did you usually eat?
In the past 7 days, how often did you eat fried tostadas/taco shells?
Each time you ate fried tostadas/taco shells, how much did you usually eat?
In the past 7 days, how often did you eat crackers?
Each time you ate crackers, how much did you usually eat?
In the past 7 days, how often did you eat popcorn?
Each time you ate popcorn, how much did you usually eat?
In the past 7 days, how often did you eat pasta/noodles?
Each time you ate pasta/noodles, how much did you usually eat?
In the past 7 days, how often did you eat tomato sauce/pasta sauce?
Each time you ate tomato sauce/pasta sauce, how much did you usually eat?
In the past 7 days, how often did you eat salsa?
Each time you ate salsa, how much did you usually eat?
In the past 7 days, how often did you eat pickled products (e.g., pickles, peppers, capers, olives, etc.)?
Each time you ate pickled products (e.g., pickles, peppers, capers, olives, etc.), how much did you usually eat?
In the past 7 days, how often did you eat leafy greens (e.g., spinach, kale, lettuce, etc.)?

Each time you ate leafy greens (e.g., spinach, kale, lettuce, etc.), how much did you usually eat? (measurements assume uncooked form)
In the past 7 days, how often did you eat seeds or nuts?
Each time you ate seeds or nuts, how much did you usually eat?
In the past 7 days, how often did you eat mushrooms?
Each time you ate mushrooms, how much did you usually eat? (The options below assume small mushrooms, not large portobello mushrooms. Assume 3 small mushrooms = 1 portobello mushrooms)
In the past 7 days, how often did you eat dried fruit?
Each time you ate dried fruit, how much did you usually eat?
In the past 7 days, how often did you eat food paste? (e.g., garlic paste, fig paste, tomato paste, chutney, etc.)
Each time you ate food paste, how much did you usually eat?
In the past 7 days, how often did you consume a single glass (5 ounces) of wine?
In the past 7 days, how often did you consume a single serving of chocolate powdered beverage mix (e.g., powders for making hot chocolate, chocolate milk, smoothies, etc.)?
In the past 7 days, how often did you consume a single serving of protein powder, mushroom powder, or other nutritional powders?
Approximately how much water do you drink each day? (including plain water, and also water added to make soups, coffee, juice mixes, smoothies, etc.)
Of the water you consume each day (including drinks, soups, etc.), what proportion is store-bought bottled water or water provided by a delivery service (e.g., 5-gallon jugs)?
Of the water you consume each day (including drinks, soups, etc.), what proportion is tap water (as opposed to bottled water or water filtered through a refrigerator or other filtration system)?
In the past 7 days, how often did you consume a single cup (8 ounces) of fruit/veggie juice?
In the past 7 days, how often did you consume a single cup (8 ounces) of tea?
If you drink more than one cup of tea in a day, do you reuse the leaves/tea bag for subsequent cups (as opposed to using a new tea bag each time)?

In the past 7 days, how often did you consume a single serving of chai powder, matcha powder, or other traditional beverage mix?
On average, how often do you add the following herbs and spices to your food? - Thyme
On average, how often do you add the following herbs and spices to your food? - Ground Sage
On average, how often do you add the following herbs and spices to your food? - Turmeric
On average, how often do you add the following herbs and spices to your food? - Italian Seasoning
On average, how often do you add the following herbs and spices to your food? - Basil
On average, how often do you add the following herbs and spices to your food? - Cumin
On average, how often do you add the following herbs and spices to your food? - Cinnamon
On average, how often do you add the following herbs and spices to your food? - Za’Atar
On average, how often do you add the following herbs and spices to your food? - Oregano
On average, how often do you add the following herbs and spices to your food? - Ginger Powder
On average, how often do you add the following herbs and spices to your food? - Ground Mustard
On average, how often do you add the following herbs and spices to your food? - Cayenne
On average, how often do you add the following herbs and spices to your food? - Rosemary
On average, how often do you add the following herbs and spices to your food? - Parsley
On average, how often do you add the following herbs and spices to your food? - Garlic Powder
On average, how often do you add the following herbs and spices to your food? - Curry Powder
On average, how often do you add the following herbs and spices to your food? - Paprika
On average, how often do you add the following herbs and spices to your food? - Masala
On average, how often do you add the following herbs and spices to your food? - Chili Powder
On average, how often do you add the following herbs and spices to your food? - Other
Each time you used a single herb/spice, how much did you usually use?
When consuming herbs and spices, do you read the serving size information displayed on the product label to determine how much of the product to use?

Of the food you consume, do you grow any of it in your home or yard?
On average, how many tree fruits (e.g., apples, oranges, avocados, etc.) of regular size do you consume from your garden per week?
On average, how many other fruits/vegetables (e.g., tomatoes, broccoli, eggplant, peppers, etc.) of regular size do you consume from your garden per week?
On average, what percentage of root vegetables (e.g., potatoes, carrots, radishes, onions, garlic, etc.) that you eat are home-grown by you?
On average, what percentage of leafy greens (e.g., spinach, kale, lettuce, etc.) that you eat are home-grown by you?
On average, what percentage of herbs/spices that you eat are home-grown by you?
Of the home-grown food you consume, what percentage is grown in a raised bed containing store-bought soil (as opposed to using dirt from your yard)?
In the past 7 days, how often did you eat candy containing either tamarind or chili powder/seasoning (e.g., Mexican tamarind candy)?
Each time you ate candy containing either tamarind or chili powder/seasoning, how much did you usually eat?
